# Evaluating the rare cases of cortical vertigo using disconnectome mapping

**DOI:** 10.1007/s00429-022-02530-w

**Published:** 2022-07-15

**Authors:** Julian Conrad, Rainer Boegle, Ria Maxine Ruehl, Marianne Dieterich

**Affiliations:** 1 Department of Neurology, Munich University Hospital, LMU Munich, Marchioninistr. 15, 81377 Munich, Germany; 2 German Center for Vertigo and Balance Disorders (DSGZ), Munich University Hospital, LMU Munich, Munich, Germany; 3 Graduate School for Systemic Neuroscience (GSN-LMU), LMU Munich, Munich, Germany; 4 Munich Cluster for Systems Neurology (SyNergy), Munich, Germany

**Keywords:** Vestibular, Insula, PIVC, Disconnectome, Corpus callosum, Cortical vertigo

## Abstract

In rare cases, cortical infarcts lead to vertigo. We evaluated structural and functional disconnection in patients with acute vertigo due to unilateral ischemic cortical infarcts compared to infarcts without vertigo in a similar location with a focus on the connectivity of the vestibular cortex, i.e., the parieto-opercular (retro-)insular cortex (PIVC). Using lesion maps from the ten published case reports, we computed lesion–functional connectivity networks in a set of healthy individuals from the human connectome project. The probability of lesion disconnection was evaluated by white matter disconnectome mapping. In all ten cases with rotational vertigo, disconnections of interhemispheric connections via the corpus callosum were present but were spared in lesions of the PIVC without vertigo. Further, the arcuate fascicle was affected in 90% of the lesions that led to vertigo and spared in lesions that did not lead to vertigo. The lesion–functional connectivity network included vestibulo-cerebellar hubs, the vestibular nuclei, the PIVC, the retro-insular and posterior insular cortex, the multisensory vestibular ventral intraparietal area, motion-sensitive areas (temporal area MT+ and cingulate visual sulcus) as well as hubs for ocular motor control (lateral intraparietal area, cingulate and frontal eye fields). However, this was not sufficient to differentiate between lesions with and without vertigo. Disruption of interhemispheric connections of both PIVC via the corpus callosum and intra-hemispheric disconnection via the arcuate fascicle might be the distinguishing factor between vestibular cortical network lesions that manifest with vertigo compared to those without vertigo.

**Supplementary Information:**

The online version contains supplementary material available at 10.1007/s00429-022-02530-w.

## Introduction

The brain continuously receives interoceptive information about the current homeostatic state, such as the position of the head and body relative to gravity via the vestibular system (Owens et al. 2018). The vestibular system is located at the crossroads between interoceptive monitoring and exteroceptive perception of the self in an allocentric space (Craig 2002; Nakul et al. 2020; Mellenthin et al. 2021). Its end organs in both ears continuously monitor and update the position of the eyes and head relative to the body and to gravity during ego- and object-motion. To provide a unique and stable perception, bilateral vestibular inputs need to be integrated with somatosensory, proprioceptive, visceroceptive and visual signals to form one common representation of the bodily Self (Angelaki and Cullen 2008; Cullen 2012). If the sensory signals from both vestibular end organs are in congruence and properly weighted, there is no conscious vestibular perception (Day and Fitzpatrick 2005).

To forward vestibular information from the labyrinth, the central vestibular brainstem pathways are bilaterally organized and reach the thalamus and the cortical network hubs via multiple crossing and non-crossing ascending tracts (Lang et al. 1979; Conrad et al. 2014; Kirsch et al. 2016) The ascending pathways of both sides mediate eye-and-head coordination in all spatial directions and the position of the head in space relative to the gravitational vector. Additionally, vestibular signals are integrated with other sensory qualities as early as on the level of the vestibular nuclei. This integrated nature ensures that bilateral multisensory signals reach the cortical vestibular network in both hemispheres (zu Eulenburg et al. 2012; Lopez et al. 2012; Frank and Greenlee 2018; Raiser et al. 2020).

An acute vestibular tone imbalance due to a unilateral peripheral or lower brainstem lesion causes severe disabling rotatory vertigo or dizziness with nausea, gait unsteadiness and falls. If lesions affect the upper parts of the central vestibular circuitry in the rostral midbrain, thalamus and cortical vestibular areas, in most instances, no rotational vertigo is reported (Anagnostou et al. 2010; Dieterich and Brandt 2015; Baier et al. 2016; Conrad et al. 2022a). This is independent of a psychophysically measurable vestibular tone imbalance that can occur such as tilts of the subjective perception of verticality (Brandt et al. 1994; Baier et al. 2016; Conrad et al. 2022a). These perceptual tilts in cortical lesions have been attributed to the higher level of sensory integration. The change of the vestibular perception in acute lesions from rotational vertigo in labyrinthine and ponto-medullary lesions to a swaying dizziness or diffuse gait imbalance can be explained by different types of vestibular neurons at brainstem and cortical level with a shift from a velocity to a position signal in the upper midbrain, thalamus and cortex (Taube 2007; Angelaki and Cullen 2008; Dieterich et al. 2018).

Whereas symptom recovery in peripheral and brainstem lesions lasts several weeks due to sensory substitution and vestibular compensation (Bronstein and Dieterich 2019; Conrad et al. 2020, 2022b), symptoms in thalamic and cortical vestibular lesions are often mild and short-lasting. One reason could be, that the contra-lesional hemisphere—where vestibular, somatosensory and visual signals are in congruence—might be able to compensate quickly for the acute vestibular cortical dysfunction suppressing the disturbing symptoms of an acute vestibular tone imbalance (Dieterich and Brandt 2015). Alternatively, the sensory weighting in cortical processing could be shifted to the unaffected sensory qualities. This could be the explanation for the results of a systematic evaluation of cortical vestibular symptoms in patients with acute ischemic hemispheric stroke that did not find vertigo symptoms and vestibular signs (Anagnostou et al. 2010).

However, in rare instances, patients with cortical infarcts suffer from cortical vertigo or dizziness mostly when a circumscribed lesion affects the parietal–opercular (retro-)insular vestibular cortex (PIVC) (Dieterich and Brandt 2015). This is also true for some instances of direct electrical stimulation of the posterior parts of the insula in patients with focal epilepsy (Penfield 1957; Mazzola et al. 2014). On the other hand, in a highly selective group of patients with small isolated infarcts restricted to the posterior insula only, no vertigo or deficits of verticality perception were reported (Baier et al. 2013). This prompted the question why in some rare cases of cortical lesions, the vestibular imbalance reaches conscious perception while in the vast majority of patients this is not the case. Could it be due to connectivity and disturbance of functional connectivity in the vestibular cortical network? Therefore, in the current study, we analyzed the pattern of disconnection in the published case reports on cortical vertigo (Dieterich and Brandt 2015). These results were compared with the lesions from the ten published patients with isolated insular infarcts without vertigo, dizziness or tilts of the SVV (Baier et al. 2013). Our hypothesis was that infarcts causing vertigo or dizziness are more densely connected to multiple hubs within the cortical vestibular network and that especially a disconnection of interhemispheric projections might lead to cortical vertigo. Usually, both hemispheres form one common spatial perception (Brandt 1997). A lesion that interrupts interhemispheric connectivity between the multisensory vestibular cortex (PIVC) of both hemispheres might suffice to disturb interhemispheric alignment of multisensory signals and thus induce cortical vertigo (Dieterich and Brandt 2015). One candidate structure could be the splenium of the corpus callosum as it contains the interhemispheric connection between both PIVCs (Kirsch et al. 2016; Wirth et al. 2018; Lemaire et al. 2021).

## Methods

### Case selection and lesion segmentation

We used the lesion figures provided in the original publications to create lesion overlap images of all ten infarcts that caused cortical vertigo provided by Dieterich and Brandt (2015) (Brandt et al. 1995; Cereda et al. 2002; Debette et al. 2003; Naganuma et al. 2006; Ahn et al. 2010; Nakajima et al. 2012; von Brevern et al. 2014; Dieterich and Brandt 2015). The infarcts were manually delineated in MNI152 space using neighboring relationships with anatomical landmarks (anterior and posterior commissures, ventricles, distal fornix, internal capsule, thalamus, and the basal ganglia) (Dieterich and Brandt 2015). Lesions were extended 2 mm from the location reported in the literature (Boes et al. 2015). For comparison, we used the lesion overlap of the ten patients with insular infarcts without vestibular deficits from Baier and colleagues (Baier et al. 2013). All lesions were delineated by an experienced neurologist (JC). A blinded (with regards to the presence of vertigo) validation of the lesion maps was carried out by RB and RMR by comparing anatomical landmarks in the original publication with the normalized lesion location. Case summaries are included as a supplementary table (Adapted from von Brevern et al. 2014; Dieterich et al. 2015; Baier et al. 2013).

### Disconnectome mapping

#### Structural disconnection

We used the tractography-based method implemented in the BCB toolkit (http://toolkit.bcblab.com/) to calculate the probability of disconnection of specific white matter tracts for each lesion (Foulon et al. 2018). High-resolution (7T) tractography data of 170 healthy participants from the Human Connectome Project (HCP) were used to track fibers that pass through each individual lesion. The patients’ lesions in MNI space are registered to the native space of each healthy participant’s tractography data using affine and deomorphic deformations (Klein et al. 2009; Avants et al. 2011). This seed is then used to perform the tractography in TrackVis (http://www.trackvis.org/). The results are transformed to visitation maps, binarized and registered to MNI152 space. From these maps, the percentage of overlap of each voxel in the normalized subject visitation maps is depicted as the voxel-wise probability of disconnection for each lesion. The number of voxels within the individual disconnectome map was thresholded at 0.5 (50% or higher probability of disconnection). In addition, we quantified the probability of disconnection using Tractotron (Thiebaut de Schotten et al. 2014). Tractotron uses the FMRIB software library (FSL; https://fsl.fmrib.ox.ac.uk/fsl/fslwiki/FSL) as well as published white matter tract atlases in the MNI152 referential to determine the pattern of disconnection induced by a lesion at the individual level (Thiebaut de Schotten et al. 2014) For each normalized lesion, tractotron gives a percentage estimate of a tract being present at this specific voxel. The probability corresponds to the lesioned voxel with the highest percentage value. A probability above 50% is considered a high probability of disconnection.

### Functional connectivity

In analogy to the structural dis-connectivity, we aimed to determine which functional network connectivities were disrupted by the lesions. While structural connectivity might be appropriate to study local connectivity of the lesion, the functional imaging approach could be more suitable for bottom-up interoceptive processes that influence multiple distant network hubs or different functional networks. With resting-state functional connectivity, spontaneous fluctuations of the BOLD signal are measured and the temporal correlation between different brain regions is considered a proxy for a functional connection. A direct structural connection is not mandatory. Therefore, distant networks which include the lesion sites could also be important for the lost function and occurrence of vertigo (Boes et al. 2015).

We used the human connectome project (HCP) “100 unrelated subjects” pre-processed resting-state fMRI dataset from the Human Connectome Project (HCP, https://www.humanconnectome.org/), Release Q3. These data contained fMRI resting-state acquisitions from 100 unrelated subjects (54 females, 46 males, mean age = 29.1 ± 3.7 years, 2 runs of 14 min 33 s each, one with phase encoding left to right and the other in the opposite direction) of the HCP 900 data release (Van Essen et al. 2012).

Data were processed following the HCP functional pre-processing guidelines (Glasser et al. 2013; Smith et al. 2013). Briefly, processing steps included artifact removal, motion correction, and registration to standard Montreal Neurological Institute space in volumetric format, with weak high pass temporal filtering (> 2000 s full width at half maximum), for slow drift removal (Smith et al. 2013). MELODIC ICA was applied to volumetric data, and artifact components were subsequently identified using FSL-FIX (Jenkinson et al. 2012; Salimi-Khorshidi et al. 2014) Artifacts and motion-related time courses (i.e., the six rigid-body parameter time series, their backward-looking temporal derivatives, plus all 12 resulting regressors squared) were then regressed out of volumetric data (Smith et al. 2013).

### Individual lesion—functional connectivity networks (FCN)

In SPM12, *t* contrasts were estimated to determine the correlations of the individual lesion–seed with the rest of the brain. Each lesion map was used as the starting point for a whole brain correlation analysis. Each lesion mask is used to create the mean time course of the region and this time course is then used for correlation with the time courses of all other voxels in the brain. All results of the functional connectivity analysis were family-wise error (*FWE*) corrected for multiple comparisons on the cluster level using threshold free cluster enhancement after calculating 10,000 permutations (Smith and Nichols 2009). Results exceeding a threshold of *p* < 0.001 FWE corrected after TFCE were considered robust against false positives. Individual results of the functional connectivity analysis are displayed with the corresponding peak *t* score intensities. To determine the common vertigo-associated lesion network, we used an overlap of eight or more fc maps for visualization.

## Results

### Lesion localization

**Fig. 1:**
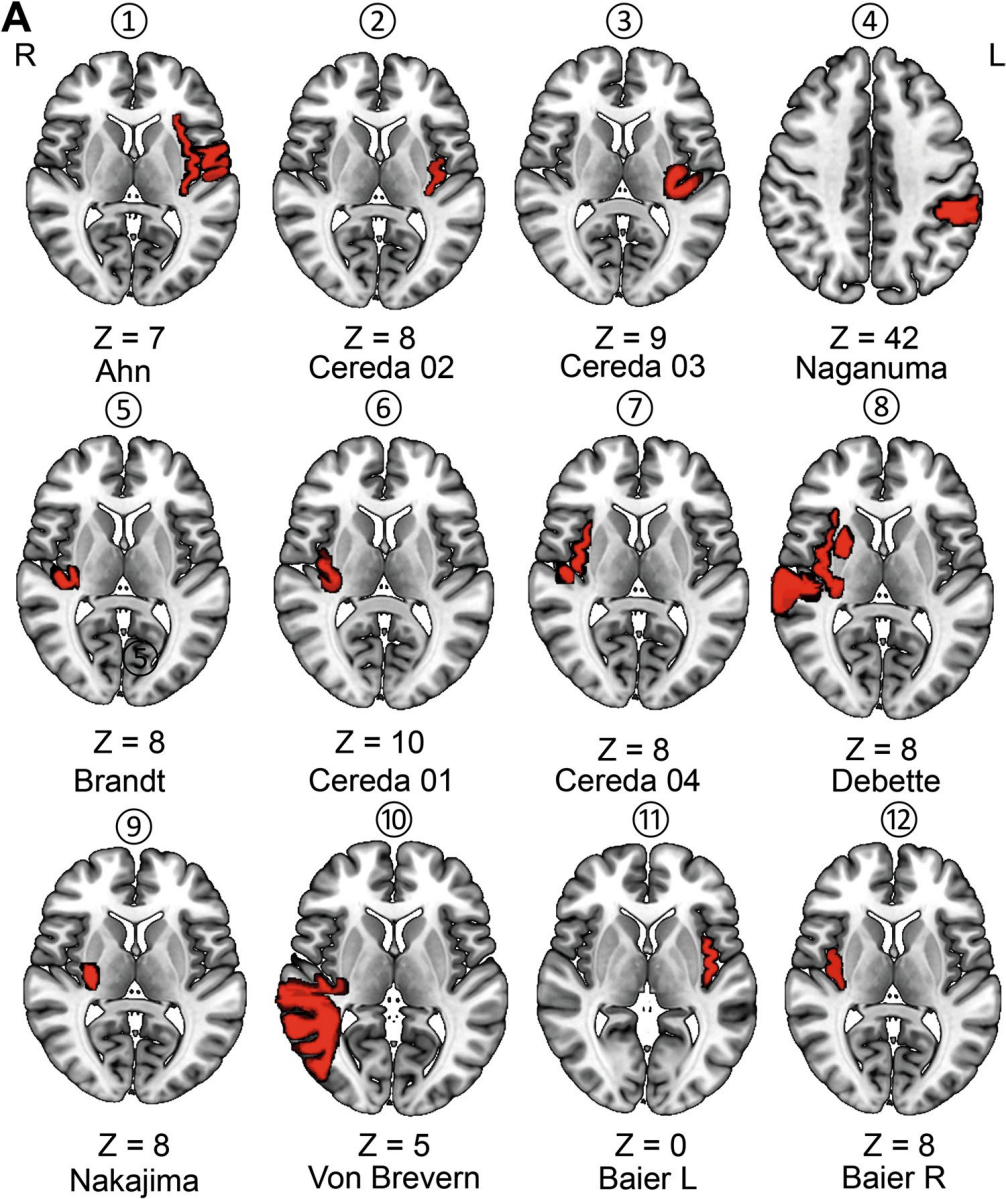
A Representative axial slices for each lesion of the published case reports on hemispheric infarcts that led to vertigo (lesions 1–10) and infarcts that did not present with vertigo (lesion overlap of 10 cases; 11–12). Left-sided lesions with vertigo are shown in the top row. Lesions were manually delineated in MNI152 space using anatomical landmark neighboring relationships

**Fig. 2:**
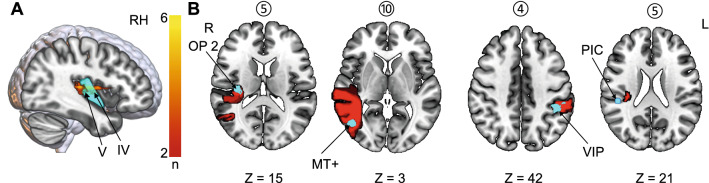
**A** Overlap image of ischemic infarcts involving the insula (overlap of at least 2 lesions is depicted): the ischemic lesions were mainly located in the posterior parts of the insula (anterior long insular gyrus and posterior long insular gyrus—IV, V). **B** In rare cases, the lesions involved vestibular structures in the parietal operculum (OP2), the intraparietal sulcus (area VIP) and the temporal lobe (MT+). None of the lesions directly affected area PIC; however, lesion 5 is in close proximity to this structure. *OP2* parietal operculum 2; *MT*+ , motion-sensitive middle temporal area; *VIP* ventral intraparietal area; *PIC* posterior insular cortex, IV anterior long insular gyrus, V posterior long insular gyrus

The ischemic lesions were centered around the insula, involving mainly the posterior parts (anterior long insular gyrus (IV) and posterior long insular gyrus (V)), and extended to the anterior insula. Representative slices for each case and the lesion overlap from (Baier et al. 2013) are depicted in Fig. 1. One lesion also covered the parietal–opercular vestibular region OP2(Brandt et al. 1995) and another one was located in the left intraparietal sulcus encompassing the vestibular multisensory area VIP (ventral intraparietal area) (Naganuma et al. 2006). Furthermore, one lesion extended toward the temporal lobe and included the motion-sensitive area MT+ (von Brevern et al. 2014). Putative cortical vestibular structures affected by the lesions are shown in Fig. 2.

### Structural disconnection

Disconnectome maps for all individual lesions were calculated using a threshold of 0.5 (> 50% probability of a tract being disconnected by the lesion). Common disconnection of all lesions (with vertigo: lesions 1–10, without vertigo: lesion overlaps 11, 12) involved the posterior parts of the fronto-insular tracts (mainly fronto-insular tracts 4 and 5) which connect the parietal operculum with the posterior parts of the insula and the inferior fronto-occipital fascicle (IFOF). The third branch of the superior longitudinal fascicle (SLF III) was also preferentially affected. Notably, two white matter tracts were disconnected in the lesions with vertigo but were spared in the lesions without vertigo (fibers of the splenium of the corpus callosum in 10/10 and posterior segments of the arcuate fascicle in 9/10 cases with vertigo). Table 1 shows the probability of specific white matter tracts being disconnected. The two white matter tracts that were disconnected in the cases with vertigo but spared in the lesions without vertigo are highlighted in bold print. The strongest claim can be made for disconnection of the CC (> 70% probability of disconnection in all ten cases with vertigo and the arcuate fascicle (> 60% probability in 7/10 and > 50% probability of disconnection in 9/10 cases). The fronto-striatal tracts were also preferentially disconnected in lesions that caused vertigo. Here, two cases had a probability of lower than 50% and two more cases just reached the probability threshold of 50%. Given the limitations of the approach based on published case reports, we did not consider this sufficient evidence for disconnection.

**Table 1:**
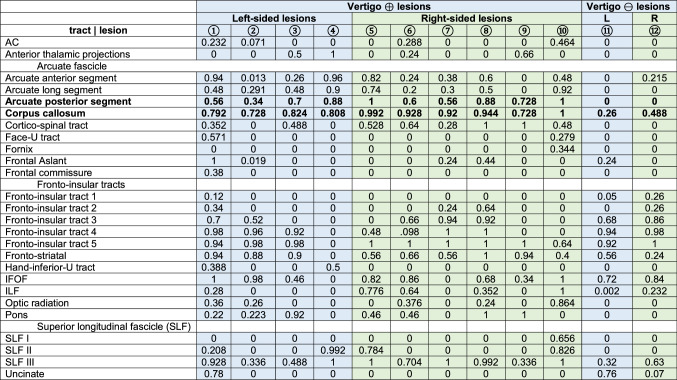
Probability of white matter disconnection for each lesion with vertigo (*n* = 10) and the lesion overlap of lesions that did not cause vertigo (*n* = 10)

*AC* anterior commissure; *IFOF* inferior fronto-occipital fascicle, *ILF* inferior longitudinal fascicle

**Fig. 3:**
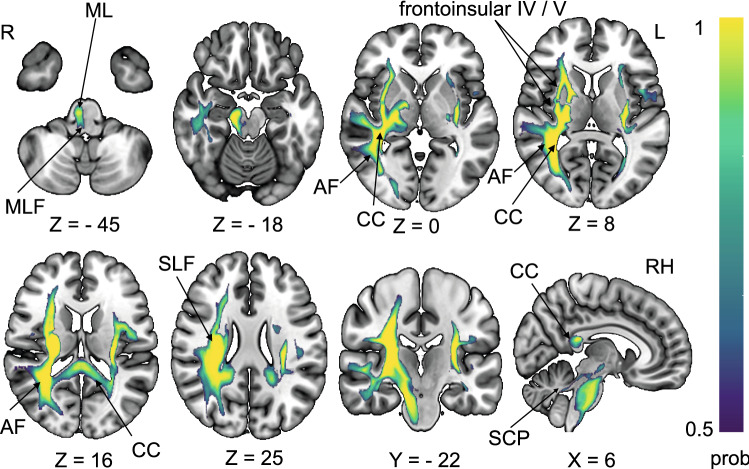
Disconnectome map of all ten lesions that manifested with vertigo. Disconnectome results are thresholded at 0.5 (> 50% probability of disconnection). Disconnection was observed for the posterior fronto-insular tracts (IV, V) that connect the posterior insula with the opercula. Intra-hemispheric disconnection affected the arcuate fascicle and the superior longitudinal fascicle (SLF, mostly third branch). Interhemispheric disconnection was observed in the splenium of the corpus callosum. Additionally, disconnection was observed with the vestibular nuclei group in the pontomedullary brainstem (possibly via the medial longitudinal fascicle and medial lemniscus) and also with the cerebellum via the superior cerebellar peduncle

Disconnection with two distinct brainstem pathways was detected connecting the pontomedullary brainstem (vestibular nuclei region) with the PIVC, one of these seemingly bypassing the thalamus while the other enters the posterolateral thalamic nuclei. In addition, disconnection was observed with the superior cerebellar peduncle. (Fig. 3, lesion disconnection of vertigo-associated lesions only). Individual disconnectome maps for each lesion are shown in the online supplement (Supplementary Fig. 1).

### Functional disconnection

**Fig. 4:**
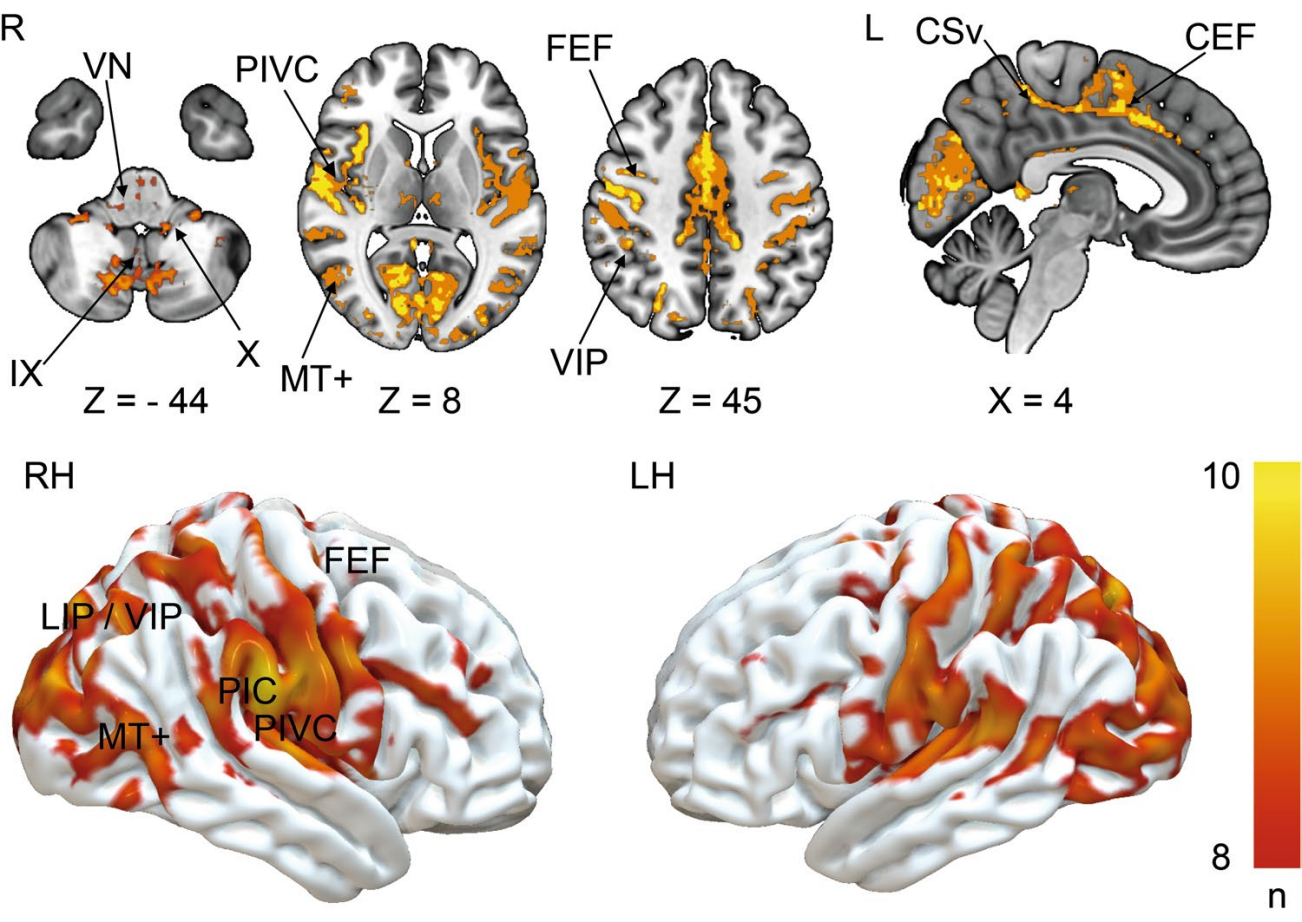
Overlap of functional connectivity networks (FCN) of the individual lesions (positive correlations with the lesion site). The individual lesion network maps are thresholded at *p* < 0.001, FWE corrected for multiple comparisons after calculating 10,000 permutations using TFCE. The figure shows an overlap of eight or more cases. Common vestibular network hubs to all lesions include the cerebellar vestibular and ocular motor representations in lobules IX (nodulus, uvula), lobule X (flocculus/paraflocculus), the vestibular nuclei (VN), the thalamus, the parieto-insular vestibular cortex (PIVC; includes insular gyri IV, V (anterior and posterior long insular gyri), area OP2, and retro-insular cortex), the posterior insular cortex (area PIC), motion-sensitive temporal and cingulate areas MT + and CSv (cingulate visual area), ocular motor and vestibular intraparietal areas LIP and VIP (lateral and ventral intraparietal area), the frontal and cingulate eye fields (FEF, CEF). The color bar gives the number of overlapping lesion FCNs

**Fig. 5:**
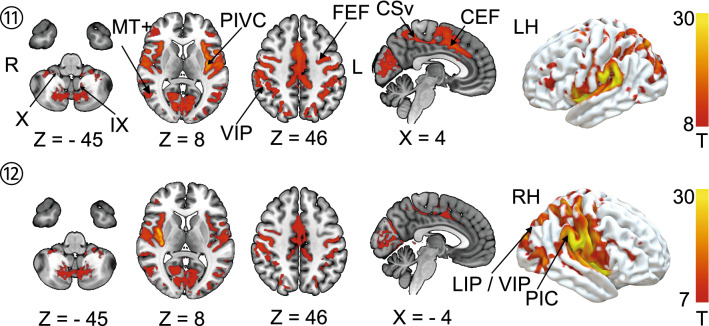
Shows the FCN of the right- and left-sided lesions of the posterior insula that did not lead to vertigo/dizziness (*n* = 10 cases). The FCN observed in these lesions was remarkably similar, involving the vestibulo-cerebellum and the cortical vestibular and ocular motor networks. All results thresholded at *p* < 0.001, FWE corrected for multiple comparisons on the cluster level after 10 000 permutations using TFCE. The color bar shows t score intensities. IX, X cerebellar lobules IX, X, MT+ motion-sensitive temporal area, *PIVC* parieto-opercular (retro-)insular vestibular cortex, *PIC* posterior insular cortex, *FEF* frontal eye field, *CEF* cingulate eye field, *LIP/VIP* lateral/ventral intraparietal area, *CsV* cingulate visual sulcus

The overlap of all individual lesion–functional connectivity network (FCN) maps was calculated to evaluate the common network structure for cortical lesions that caused vertigo. Figure 4 shows the overlap of eight or more FCNs in the group with vertigo. The individual lesion FCNs are shown in Supplementary Fig. 2. The common subcortical components of the FCNs include the vestibular nuclei (VN), the cerebellar vestibular and ocular motor representations in lobules IX (nodulus, uvula) and X (flocculus/paraflocculus). Cortical network hubs included the PIVC, bordering posterior insular cortex (PIC) and adjacent superior temporal gyrus but also more distant vestibular multisensory areas, such as the ventral intraparietal area (VIP), motion-sensitive areas MT+ in the temporal lobe and cingulate visual sulcus (CSv) and ocular motor areas of the parietal (lateral parietal area—LIP) and frontal lobes (frontal eye fields, FEF, and dorsolateral prefrontal cortex, DLPFC). Additional connectivity was also seen with primary somatosensory and visual cortex. However, when the seed of the lesion without vertigo overlaps was considered individually (lesions 11, 12), a strikingly similar network emerged (Fig. 5).

## Discussion

The main findings of this lesion disconnectome study were as follows: (i) Lesions that caused vertigo compared to those without vertigo disconnected parts of the cortical vestibular network and interhemispheric connections via the corpus callosum and the posterior parts of the arcuate fascicle. (ii) A common lesion FCN involved cerebellar lobules IX, X, the vestibular nuclei, the thalamus, the PIVC (anterior/posterior long gyrus, retro-insular cortex, OP2), posterior insular cortex (area PIC), parietal multisensory vestibular area VIP, the parietal, frontal and cingulate eye fields (area LIP, FEF, CEF), motion-sensitive areas of the temporal lobe (MT+) and cingulate cortex (CSv). However, the identification of this network did not seem sufficient to explain cortical vertigo as these structures were also part of the lesion FCN of patients without vertigo.

### Lesion functional connectivity mapping

The individual lesion FCNs emphasized the network aspect of the cortical vestibular system (Bense et al. 2001; Dieterich et al. 2003; Raiser et al. 2020) The functional connectivity showed involvement of the VN, the cerebellar vestibular lobules IX, X and the cortical vestibular and multisensory areas (PIVC (insular gyri IV, V, OP2, Ri), area PIC, area VIP, MT + , CSv) (Raiser et al. 2020). Fittingly, we found functional connectivity with components of the cortical ocular motor centers in the cerebellum, parietal, cingulate and frontal lobes (ocular motor vermis (OMV), areas LIP, CEF, FEF). A close interaction of the vestibular and ocular motor system is known, as demonstrated in existing imaging evidence (Dieterich et al. 2009; zu Eulenburg et al. 2012).

Interestingly, the patterns of the established FCNs did not substantially differ between the lesions that led to cortical vertigo compared to those that did not. Therefore, a lesion FCN difference based only on the location and extent of the lesions was not sufficient to explain the differences between lesions with vertigo and those without vertigo.

### White matter disconnection

Using disconnectome mapping, we identified two distinct white matter structures that were lesioned in 10/10 (CC) and 9/10 (AF) cases with vertigo but were spared in the lesion overlaps of cases without vertigo.

### Posterior segment of the arcuate fascicle

The arcuate fascicle is part of the superior longitudinal fiber system that comprises the SLF (I–III) and the arcuate fascicle (Vavassori et al. 2021). It connects the inferior, middle and superior temporal gyri and the supra-marginal gyrus with the frontal operculum (pars opercularis, pars triangularis), middle frontal gyrus (MFG) and the ventral premotor cortex and is left-lateralized (Fernández-Miranda et al. 2015). Its posterior portion connects the temporal parts with the supra-marginal gyrus (SMG) and angular gyrus (AG) of the inferior parietal lobule (IPL).

While these tracts run in close proximity to the insular and parietal–opercular cortex, terminations in these regions seem not to exist. In terms of function, the left AF is recognized as part of the language network (Forkel et al. 2011, 2020). The function of the right AF is far less well-defined but has been implicated in the ability to recognize the mental state of others (Herbet et al. 2014). A role of the AF in vestibular processing has not been proposed up to now. Additionally, the probability for disconnection was lower in the cases with vertigo compared to the CC. A theoretical framework to explain the affection of the AF does not exist yet. In our opinion, this might be an epiphenomenon of lesions that affect the PIVC and underlying white matter.

### Corpus callosum

The CC is the largest white matter tract of the human brain and is the main interhemispheric connection in humans. Parcellations of the CC have been proposed on anatomical landmarks, histology, structural connectivity, functional connectivity gradients and simple geometry (Witelson 1989; Aboitiz and Montiel 2003; Hofer and Frahm 2006; Friedrich et al. 2020). The anterior and mid-portions project to the prefrontal and motor cortex, while the more posterior regions connect the temporal, parietal and occipital lobes. An interhemispheric connection of the PIVC has been described by DTI in humans via the splenium of the CC and might be involved in the reorganization of verticality perception after ischemic infarcts. (Kirsch et al. 2016; Wirth et al. 2018; Lemaire et al. 2021). Indeed, in patients with subcortical ischemic strokes presenting with vestibular symptoms, vestibular recovery/compensation took place over time and was accompanied by volumetric changes in the splenium of the CC using voxel-based morphometry (Conrad et al. 2022a, b). In addition, volumetric increases were also seen in the white matter around the PIVC after recovery of perceptual deficits, i.e., tilts of the SVV (Conrad et al. 2022b). These volumetric increases of the CC also map to the interhemispheric connection between the PIVCs of both hemispheres.

### Interhemispheric connectivity of the PIVC

Based on the current data, we provide evidence for the theory that interhemispheric inhibitory connections from the contra-lesional (intact) PIVC can suppress the perception of vertigo (Dieterich and Brandt 2015). Visual and vestibular inputs have to be matched for the proper detection of the position of head and body in space relative to the gravitational vector to provide a congruent perception of position and motion in space. In healthy individuals, there are no diverging visual and vestibular perceptions (Brandt 1997). In the case of lesions to the PIVC, the mismatch between vestibular and visual inputs could be detected by a central predictor via a feedback loop connecting both hemispheres where the predicted input is compared with the actual sensory input (Brandt et al. 2005). The visual–vestibular signal which best matches the predicted input then is further processed while the input from the lesioned hemisphere is suppressed. In contrast, if the interhemispheric connection between the PIVCs is disturbed, the intra-hemispheric visual–vestibular mismatch and/or the interhemispheric mismatch is perceived as cortical vertigo.

### Limitations

The current study was based on case reports of rare patients with cortical infarcts that led to vertigo. In these publications, single slices depicting the lesions are presented. The constructed lesion maps are approximations of these lesions. Additionally, we were not able to obtain the original lesion maps of the control group. Therefore, the maximum lesion overlap presented in the figures of the original paper was used for left- and right-sided lesions that did not cause vertigo. The lesion delineation procedure has been previously reported to provide reliable results (Boes et al. 2015; Darby et al. 2018; Cohen et al. 2019). Due to the number of cases and unbalanced groups (ten vs. two lesion maps), statistical group comparisons were not carried out. The lesion network mapping approach uses individual lesions as seed regions for the structural and functional connectivity analysis in healthy individuals. The large number of healthy subjects and the methodology help to mitigate the influence of inter-individual differences in connectivity. However, it is still possible that the disconnectome and fc maps do not reflect the individual connectivity profiles of the patients. Given these limitations, the findings have to be interpreted with caution.

In our opinion, the current results provide the most accurate evidence for interhemispheric disconnection in these patients with cortical infarcts to date. Because of the rarity of cortical vertigo, a systematic prospective evaluation of cortical vertigo was not helpful to answer this particular research question (Anagnostou et al. 2010).

It is possible that subcomponents of the lesion FCN between lesions that caused vertigo vs. those that did not might differ. A more fine-grained voxel-by-voxel analysis of connectivity differences might decipher subtle FCN differences both groups (Kirsch et al. 2018).

## Conclusion

A structural disconnection of the splenium of the CC and the arcuate fascicle might be the distinguishing factor between lesions that caused vertigo (structural interhemispheric disconnection) and lesions that did not (CC and AF spared). Infarcts that caused vertigo and lesions that did not lead to vertigo are part of the same functional connectivity network that links common subcortical and cortical vestibular hubs. Therefore, the lesion FCN seems not sufficient to explain the occurrence of cortical vertigo.

## Supplementary Information

Below is the link to the electronic supplementary material.

Supplementary file1 Representative slices of individual disconnectome maps for each lesion. The individual disconnectome maps were thresholded at 0.5 (> 50% probability of disconnection) (EPS 58668 KB)

Supplementary file2 Case summaries of the individual cases and summary of the clinical findings from Baier et al. 2013. (Adapted from Dieterich et al. 2015, Baier et al. 2013, von Brevern et al. 2014) (DOCX 18 KB)

Supplementary file3 Representative slices of the lesion FCN for each individual lesion. All results were thresholded at p<0.001, FWE corrected for multiple comparisons after non-parametric permutation testing (TFCE) calculating 10000 permutations. Results are depicted with the T score intensities corresponding to the threshold after permutation testing (EPS 106584 KB)

## Data Availability

The individual lesion and disconnectome maps will be publicly available upon publication (https://osf.io/tr8x2/).
