# Supplementary material for: Evaluating the rare cases of cortical vertigo using disconnectome mapping
Source: Brain Struct Funct. 2022 Jul 15;227(9):3063–73. doi: 10.1007/s00429-022-02530-w (PMC9653368; doi:10.1007/s00429-022-02530-w)
Supplement: Supplementary file 2 — Supplementary file2 Case summaries of the individual cases and summary of the clinical findings from Baier et al. 2013. (Adapted from Dieterich et al. 2015, Baier et al. 2013, von Brevern et al. 2014) (DOCX 18 KB) [file 429_2022_2530_MOESM2_ESM.docx]

| **Lesion (main author)*** | **s / age (y), n** | **vestibular subjective symptoms** | **vestibular objective symptoms** | **duration** | **non-vestibular symptoms** | **localization** | **Lesion vol (cc)** |
| --- | --- | --- | --- | --- | --- | --- | --- |
| **①** Ahn et al. | ♀ / 51 | vertigo | horizontal / torsional SPN R, HSN, 18% side difference calorics left | 10 d  + 1-2 min attacks for 9 months | word finding difficulties | insula, frontal operculum, superior temporal gyrus | 6.75 |
| **②** Cereda et al. #02 | ♂ / 69 | dizziness, lateropulsion, falls | none | 1 week | hemihypesthesia with impaired position sense, fluent aphasia, dysarthria, taste dysfunction | posterior insular short gyrus, anterior / posterior insular long gyrus | 0.57 |
| **③** Cereda et al. #03 | ♀ / 48 | Sudden sense of heaviness of the right hemibody | none | 2 days | sensory deficit (touch, pain, temperature, vibration, postural sense), non-fluent aphasia, dysarthria | anterior / posterior insular long gyrus, Heschl gyrus, Ri, extreme capsule, putamen, claustrum | 2.32 |
| **④** Naganuma et al. | ♀ / 65 | rotational vertigo (head movement dependent) | SPN R | max. 3 days | none | Intraparietal sulcus (AIP, LIP, VIP), IPL (PF) | 4.99 |
| **⑤** Brandt et al. | ♀ / 30 | rotational vertigo, falls | SVV tilt -7° | 1 week | hypesthesia | anterior / posterior insular long gyrus, area OP2, area PGp | 4.66 |
| **⑥** Cereda et al. #01 | ♀ / 73 | dizziness, non-rotational vertigo, gait unsteadiness | none | 3 days | none | anterior / posterior insular long gyrus, Heschl gyrus, Ri, | 2.28 |
| **⑦** Cereda et al #04 | ♀ / 75 | dizziness, instability | none | 2 weeks | alien limb phenomenon, hypesthesia (pain, touch) | posterior insular short gyrus, anterior / posterior insular long gyrus, Ri Heschl gyrus | 1.98 |
| **⑧** Debette et al. | ♂ / 51 | rotational vertigo | 1. gaze-evoked nystagmus R  2. head / body tilt R | 1. < 30 min  2. 2 months | hemiplegia, hemihypesthesia (touch, vibration) | putamen, thalamus, anterior / posterior insular short gyrus, anterior / posterior insular long gyrus, Heschl gyrus, Ri, STG | 5.74 |
| **⑨** Nakajima et al. | ♂ / 57 | rotational vertigo | SPN L | days (truncal ataxia > 2 months) | hemiparesis, truncal ataxia | putamen, internal, external, extreme capsule, claustrum | 1.89 |
| **⑩** vonBrewern et al. | ♀ / 51 | rotational vertigo, nausea, imbalance | horizontal rotatory SPN R, SVV tilt + 3.2° | days | visual field defect (left lower quadrant), spatial neglect (visual) | STG / MTG / ITG (includes MT+) Heschl gyrus, Ri | 31 |
| **⑪** Baier et al. (sum L) | 4/5 ♀ /  74 (+/-10) | none | none | n. a. | aphasia (4/5), dysarthria (2/5) | anterior, middle, posterior insular short gyrus, anterior / posterior insular long gyrus, | 2.16 |
| **⑫** Baier et al. (sum R) | 3/5 ♀ /  69 (+/-8) | none | none | n. a. | dysarthria, somatosensory deficits (2/5 each) | posterior insular short gyrus, anterior / posterior insular long gyrus | 1.67 |
| SPN spontaneous nystagmus, HSN head shaking nystagmus, SVV subjective visual vertical, R right, L left, Ri retroinsular cortex, AIP anterior intraparietal area, LIP lateral intraparietal area, VIP ventral intraparietal area, IPL inferior parietal lobule, PF inferior parietal lobule, supramarginal gyrus subdivision, OP2 parietal opercular cortex 2, PGp angular gyrus, STG superior temporal gyrus, MTG middle temporal gyrus, ITG inferior temporal gyrus, MT+ motion sensitive temporal area, cc cubic centimeters y (years), s (sex), n number.  *adapted from Dieterich et al. 2015, Baier et al. 2013, von Brewern et al. 2014 | | | | | | | |
